# Preovulatory exposure to a protein-restricted diet disrupts amino acid kinetics and alters mitochondrial structure and function in the rat oocyte and is partially rescued by folic acid

**DOI:** 10.1186/s12958-019-0458-y

**Published:** 2019-01-17

**Authors:** Amy K. Schutt, Chellakkan S. Blesson, Jean W. Hsu, Cecilia T. Valdes, William E. Gibbons, Farook Jahoor, Chandra Yallampalli

**Affiliations:** 1 Division of Reproductive Endocrinology and Infertility, Department of Obstetrics and Gynecology, Baylor College of Medicine, Houston, TX USA; 2 USDA/ARS Children’s Nutrition Research Center, Department of Pediatrics, Baylor College of Medicine, Houston, TX USA; 3 Texas Children’s Hospital Pavilion for Women, 6651 Main St, Suite F1020, Houston, TX 77030 USA

**Keywords:** Oocyte, Developmental programming, Amino acid, Metabolism, Protein restriction, Mitochondria, Kinetics

## Abstract

**Background:**

Detrimental exposures during pregnancy have been implicated in programming offspring to develop permanent changes in physiology and metabolism, increasing the risk for developing diseases in adulthood such as hypertension, diabetes, heart disease and obesity. This study investigated the effects of protein restriction on the metabolism of amino acids within the oocyte, liver, and whole organism in a rat model as well as effects on mitochondrial ultrastructure and function in the cumulus oocyte complex.

**Methods:**

Wistar outbred female rats 8–11 weeks of age (*n* = 24) were assigned to three isocaloric dietary groups, including control (C), low protein (LP) and low protein supplemented with folate (LPF). Animals were superovulated and 48 h later underwent central catheterization. Isotopic tracers of 1-^13^C-5C^2^H_3_-methionine, ^2^H_2_-cysteine, U-^13^C_3_-cysteine and U-^13^C_3_-serine were administered by a 4 h prime-constant rate infusion. After sacrifice, oocytes were denuded of cumulus cells and liver specimens were obtained.

**Results:**

Oocytes demonstrated reduced serine flux in LP vs. LPF (*p* < 0.05), reduced cysteine flux in LP and LPF vs. C (*p* < 0.05), and a trend toward reduced transsulfuration in LP vs. C and LPF. Folic acid supplementation reversed observed effects on serine flux and transsulfuration. Preovulatory protein restriction increased whole-body methionine transmethylation, methionine transsulfuration and the flux of serine in LP and LPF vs. C (*p* = 0.003, *p* = 0.002, *p* = 0.005). The concentration of glutathione was increased in erythrocytes and liver in LP and LPF vs. C (*p* = 0.003 and *p* = 0.0003). Oocyte mitochondrial ultrastructure in LP and LPF had increased proportions of abnormal mitochondria vs. C (*p* < 0.01 and *p* < 0.05). Cumulus cell mitochondrial ultrastructure in LP and LPF groups had increased proportions of abnormal mitochondria vs. C (*p* < 0.001 and *p* < 0.05). Preovulatory protein restriction altered oocyte expression of Drp1, Opa-1, Mfn1/2, Parl and Ndufb6 (*p* < 0.05) and Hk2 (*p* < 0.01), which are genes involved in mitochondrial fission (division) and fusion, mitochondrial apoptotic mechanisms, respiratory electron transport and glucose metabolism.

**Conclusions:**

Preovulatory protein restriction resulted in altered amino acid metabolism, abnormal cumulus oocyte complex mitochondrial ultrastructure and differential oocyte expression of genes related to mitochondrial biogenesis.

## Background

Prenatal exposure to aberrant conditions such as protein restriction in the rat, high fat diet, maternal diabetes, maternal obesity, and variable stress in the mouse, and famine in humans, have been implicated in programming mammalian offspring to develop permanent changes in physiology and metabolism, increasing the risk for developing diseases in adulthood such as hypertension, diabetes, heart disease and obesity [1–10]. The increased susceptibility is the result of fetal adaptations in utero in response to altered nutrient availability [11–13]. This investigation explores the ovary as a postulated target vulnerable to malnutrition, representing a window of time prior to conception when adaptations to altered nutrient availability may impact future pregnancies [14–16]. In this model the oocyte must adapt to maternal exposures, potentially predisposing future embryos, and thus offspring, to developmental abnormalities.

Maternal dietary protein restriction has been shown to induce significant changes in embryos and offspring in animal models [5, 17, 18]. Given 3–4 weeks prior to pregnancy, a low-protein (LP) diet results in altered mitochondrial metabolism in 2-cell embryos [19]. Given during the preimplantation period, a LP diet decreases the rate of blastocyst cellular proliferation, reduces maternal serum levels of essential amino acids, and induces intrauterine growth restriction and hypertension in pups [20]. Given during pregnancy, a LP diet results in insulin resistance in adult male offspring as well as epigenetic modifications in hepatic gene expression [21]. These hepatic epigenetic changes are prevented with folate supplementation [22, 23]. Epigenetic modifications include DNA methylation at CpG sites and modification of chromatin. CpG sites are DNA regions consisting of cytosine and guanine nucleotides joined by a phosphate group whereby epigenetic modification occurs when the cytosine is methylated changing the expression of a gene. The mechanism by which folic acid reverses these observed epigenetic changes is via its action as a single carbon donor. The metabolic pathway responsible for the production of single carbon groups is closely linked to folic acid metabolism. Hence, folic acid modulates the expression of DNA methyltransferases which in turn has been shown to ameliorate altered DNA methylation due to maternal undernutrition. It is recommended that women of reproductive age consume at least 400 mcg of folic acid prior to and during pregnancy.

A gestational LP diet causes insulin resistance in adult male offspring via an inefficient insulin-induced insulin receptor, as well as impaired GLUT-4 translocation [24]. GLUT-4 is the insulin-regulated glucose transporter that is located in adipose, skeletal and cardiac tissues. It has therefore been proposed that one-carbon metabolism is central to the mechanism of this altered phenotype [25–27]. These experiments investigated folate supplementation in the reproductive aged female rat as a potential means to ameliorate potential adverse effects on the oocyte due to dietary protein deficiency [28].

The oocyte requires an enormous supply of energy both during maturation and after fertilization to support critical events such as the resumption of meiosis, spindle formation and cellular division [29]. Mitochondria are maternally-inherited organelles within the oocyte responsible for the production of the chemical energy precursor adenosine triphosphate (ATP) via oxidative phosphorylation [30]. Reactive oxygen species (ROS) can damage mitochondria if not appropriately neutralized by antioxidants such as glutathione (GSH). The formation of GSH is dependent upon the availability of precursors such as folate, serine and cysteine. In this study oocyte kinetics of serine and cysteine were evaluated to evaluate the process of transsulfuration, which results in the production of cysteine (rate-limiting precursor to the formation of GSH) (Table 3). This study also sought to assess the effect of protein restriction on the concentration of amino acids within cumulus cells (Table 3). Mitochondria also have key roles in maintaining cellular homeostasis and regulating apoptosis [31]. Importantly, embryonic mitochondrial replication does not occur until after blastocyst hatching and implantation, and the existing pool of mitochondria available to each cell of the developing embryo diminishes with each successive cellular division [32, 33]. Sufficient ATP production by mitochondria is therefore required for successful nuclear and cytoplasmic maturation of the oocyte [34].

Oocyte mitochondria from mice on a high fat diet or from mice with diabetes demonstrate disarrayed cristae, inner mitochondrial membrane swelling, and increased vacuoles within the organelle [15, 35]. Additionally, under physiologic conditions, mitochondrial morphology is in part maintained by a balance of fusion and fission [36, 37]. Mitochondrial fusion and fission are actions that fuse and divide, respectively, the two lipid bilayers that encompass mitochondria [36, 37]. DRP-1 is a molecule that regulates mitochondrial fission [38]. Cellular starvation causes phosphorylation of *DRP1*, decreasing its recruitment to mitochondria and therefore inhibiting fission [39]. Similarly, mouse embryonic fibroblasts exposed to various cellular stressors such as ultraviolet radiation or actinomycin D causes stress-induced mitochondrial hyperfusion (SIMH) [40]. Mitochondrial elongation can increase the ATP synthesis capacity of each mitochondrion [41].

**Fig. 1:**
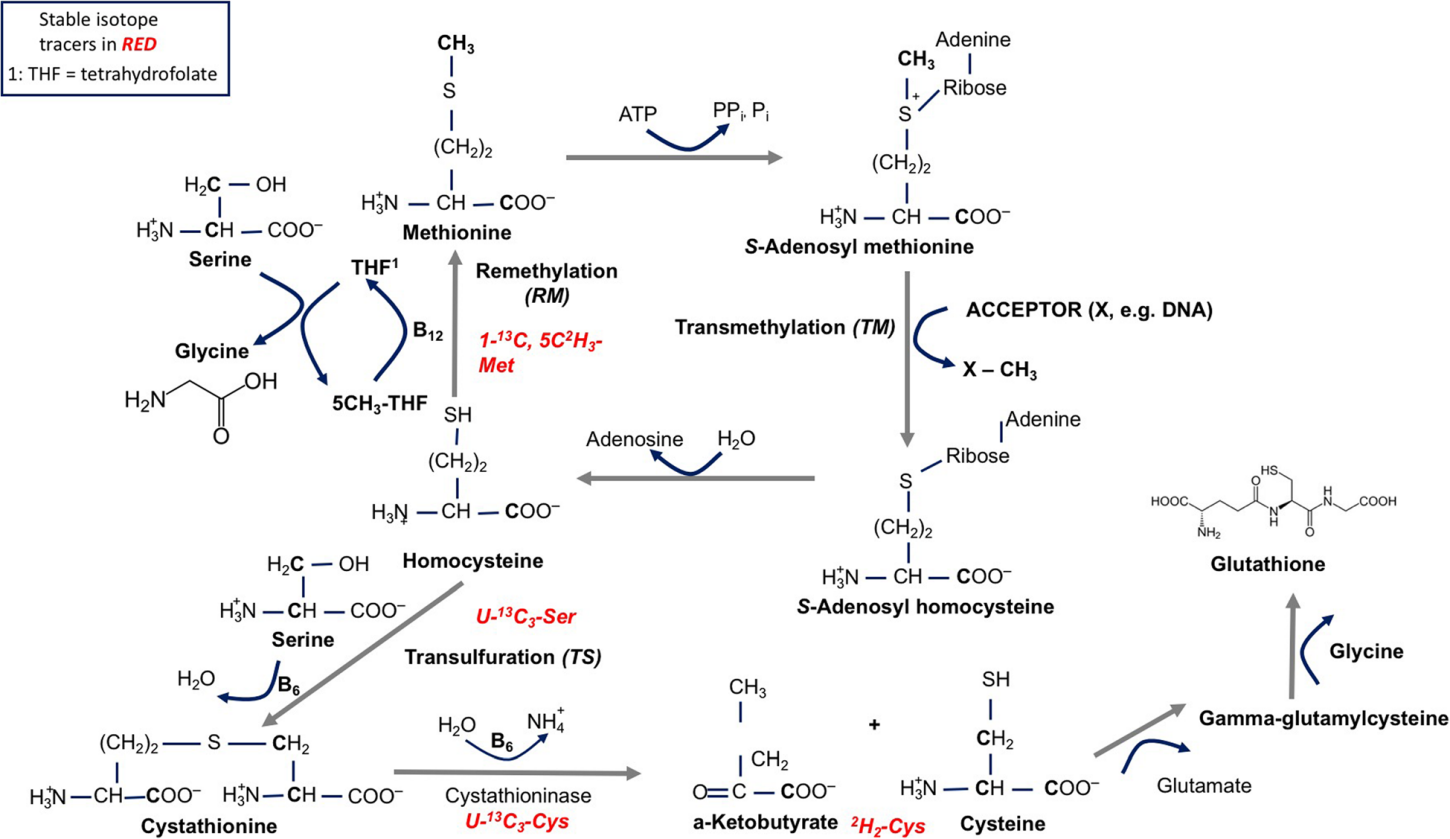
Transmethylation and transsulfuration pathways demonstrating methionine and homocysteine metabolism

Dietary protein restriction may reduce the intracellular production of antioxidants capable of detoxifying local ROS produced by mitochondria via altered amino acid metabolism (Fig. 1). The primary intracellular antioxidant is glutathione, which is capable of preventing cellular damage by serving as an electron donor via the reduction of disulfide bonds. Glutathione is a reducing agent, and once an electron has been donated it is converted to its oxidized form, glutathione disulfide (GSSG). GSSG can be reduced back to GSH via glutathione reductase. Figure 1 illustrates the transmethylation and transsulfuration pathways. Integral to these pathways is the regulation of the transfer of methyl groups for the synthesis of metabolic intermediates and the methylation of DNA, RNA and proteins. Serine and glycine are nutritionally non-essential amino acids but are critical for proper cell function. Serine provides methyl groups to tetrahydrofolate (THF) to form 5-methyl-THF, which is an intermediate in the conversion of homocysteine to methionine. Therefore, the availability of methionine is in part a reflection of resynthesis from homocysteine. The methylation of homocysteine to methionine requires an adequate supply of folic acid (which is twice reduced to THF) [42].

A low-protein diet may alter the kinetics of whole-body and tissue-specific amino acid metabolism. In addition to resynthesis from homocysteine, methionine, a nutritionally essential amino acid in humans, is obtained through dietary intake and released from protein breakdown [43, 44]. Methionine is a critical intermediary in the production of glutathione, the primary intracellular antioxidant, and improving cellular access to methionine may improve alterations in metabolism that occur as a result of protein deficiency. Therefore, folic acid supplementation may ameliorate decreased methionine availability by improving the efficiency of RM of homocysteine to methionine, as folate acts as a methyl donor to support the remethylation of homocysteine to methionine [45]. Methionine is the sulfur donor for cysteine synthesis, where cysteine is the rate-limiting precursor for the synthesis of glutathione, the primary intracellular antioxidant [44].

The liver is a digestive organ that is essential for the synthesis of proteins, regulation of glycogen storage, and detoxification of metabolites, among other important metabolic roles [46]. Normal hepatic function necessitates proper amino acid metabolism, and in humans approximately 50% of methionine is metabolized to S-adenosylmethionine (SAMe) by the liver. In turn, SAMe is utilized for the methylation of substrates and for protein synthesis [47]. Therefore, studying amino acid metabolism in metabolically-active tissues (including the liver and the oocyte) in a low protein environment presents a more complete picture of the whole body metabolic effects. Thus a low-protein diet, when given before conception, may reduce oocyte amino acid concentrations through altered amino acid kinetics, restricting the availability of substrates needed for the protection, optimal growth, and fertilization of oocytes. Additionally, Our hypothesis is that preovulatory exposure to a protein-restricted diet alters the metabolism of amino acids and augments mitochondrial ultrastructure and function in the rat cumulus oocyte complex.

## Methods

Baylor College of Medicine Institutional Review Board (IRB) and the Institutional Animal Care and Use Committee (IACUC) approved this protocol. The isocaloric diets used in this study were customized through Teklad Laboratory Animal Diets (Envigo), and included a control group with a normal amount of protein (20% casein), a low protein group (6% casein), and a low protein plus supplemental folate group (6% casein plus 5 mg/kg folate) [5]. The recommended daily allowance (RDA) for rats is approximately 60 kcal with 20% protein and 1 mg/kg folic acid. The RDA for protein and folic acid were utilized for development of the control diet. The caloric requirement during pregnancy in the rat is 10 to 30% greater than that of the mature, nonpregnant female rat. [48] The protein-derived calories that were reduced in the low protein groups were replaced by carbohydrate sources. 8–9 week old virgin female Wistar rats (200 g) were randomized to three groups (*n* = 8 each) for a 40-day isocaloric dietary intervention. The length of dietary intervention was chosen because the length of the estrous cycle in a rat is approximately 4 to 5 days, and 40 days represents approximately 8 estrous cycles in the rat. In the human, primordial follicles leave the resting state 26 weeks prior to ovulation, and the most extensive follicular growth (from primary to late tertiary) occurs from around 14 weeks preovulation [27]. During this time the oocyte grows 60–70 fold in size and undergoes rapid cellular biosynthesis. Therefore, 40 days in the rat represents the length of time needed to expose primordial follicles throughout their growth and maturation until they become preovulatory follicles.

### Amino acid kinetics

At the conclusion of the dietary intervention the animals were injected with 15 U of PMSG (pregnant mare serum gonadotropin) in preparation for the amino acid stable isotope infusion. Animals were fasted for 12 h prior to carotid and jugular catheter implantation on the morning of the infusion experiment. Central catheterization was performed as previously described [49]. In order to provide an understanding of methionine homeostasis in a low protein environment, the metabolism of methionine and homocysteine were investigated via a stable isotope infusion protocol using the tracer dilution method. Kinetic parameters of the methionine (one-carbon) cycle were measured including transmethylation (TM), remethylation (RM), and transsulfuration (TS) [44, 50–52].

Stable isotope-labeled amino acids (1-^13^C-5C^2^H_3_-methionine, ^2^H_2_-cysteine, U-^13^C_3_-cysteine and U-^13^C_3_-serine), were administered as a primed bolus and then as a continuous infusion, through jugular vein catheters for 4 h. A prime dose of U-^13^C_3_-cysteine was also given to prime the secondary pool of cysteine with labels derived from U-^13^C_3_-serine. Timed blood samples were taken during the infusion followed by harvesting of tissues and oocytes, which allowed for the calculation of the metabolic flux, or rate of turnover of amino acids in the whole body and within the oocyte. The plasma or tissue isotope enrichments of methionine, homocysteine, cysteine, serine and glutathione were measured by liquid chromatography tandem mass spectrometry (LC-MS/MS) as previously described by us [44, 51]. The fluxes of methionine, cysteine, serine and methionine TM, TS and RM rates were calculated using standard steady state equations as previously described [44, 50–52].

In order to understand GSH homeostasis in a low protein environment, both with and without folate supplementation, GSH kinetics and concentration were measured in vivo. GSH synthesis rate was measured using the rate of incorporation of ^2^H_2_-cysteine and the precursor/product equation. To measure the isotopic enrichment of erythrocyte and liver tissue glutathione, liver tissue and RBC-free extract was first reduced by adding a 0.5 M dithiothreitol (DTT) solution, and then was analyzed by LC-MS/MS. Erythrocyte and liver tissue glutathione concentrations were measured by an isotope dilution method. Briefly, a known amount of sample was spiked with a known quantity of labeled glutathione-[glycine-^13^C_2_,^15^N] (Cambridge Isotope Laboratories, Tewksbury, MA) as an internal standard. The samples were then analyzed by LC/MS and concentrations calculated as previously described [44].

### Cumulus oocyte complex mitochondrial ultrastructure

Twenty-four ovarian specimens were processed (*n* = 8 for each of the three experimental groups) for transmission electron microscopy (TEM) as previously described. Once an antral follicle with an intact cumulus oocyte complex (COC) was identified at 40x magnification, the ovarian tissue block was cut into thin (50 nm) sections by the ultramicrotome. These thin sections were placed on a copper grid and stained with heavy metals. This produced mounted tissue specimens that were available for imaging under the electron beam of the TEM.

One antral follicle was imaged from each of 12 animals, with four animals in each of the three experimental groups. The COCs were too large to fit wholly within one grid box. Therefore, each antral follicle was analyzed as follows in a systematic and semi-quantitative manner. Three random fields of each oocyte were imaged at 2500x magnification, and three random fields of cumulus cells from each COC were imaged at 2500x magnification. The number of mitochondria that were visualized in each of the fields at 2500x was tallied.

One thousand three hundred forty oocyte mitochondria and 1708 cumulus cell mitochondria were imaged and analyzed in a qualitative fashion. Cumulus and oocyte mitochondria were stratified by their qualitative appearance as normal or abnormal. Normal mitochondria in germinal vesicle stage oocytes appear spherical or oval in shape with a dense matrix, well-organized cristae, and clearly delineated inner and outer mitochondrial membranes. Normal mitochondria are not elongated, do not have vacuoles or inclusions, and do not have swollen inner mitochondrial membrane cristae. Cumulus oocyte mitochondria were imaged at 2500x magnification and categorized by their subjective appearance regarding the aforementioned criteria that represent normal and abnormal mitochondria.

### Oocyte mRNA expression

The Qiagen REPLI-g WTA (whole transcriptome amplification) kit was used for whole transcriptome amplification of total RNA from small cohorts of denuded oocytes that were used for the creation of a cDNA library. The REPLI-g amplified cDNA library was stored at − 20 °C for the downstream application of real-time qPCR. The cDNA library was diluted to 1:100, at a volume of 2–3 μl diluted DNA for each 20 μl real-time PCR reaction volume. The Quantiscript RT mix, ligation mix, and REPLI-g SensiPhi amplification mix were always prepared fresh. All buffers and reagents were vortexed before use to ensure thorough mixing. The Quantiscript RT Enzyme Mix, Ligase Mix, and REPLI-g SensiPhi DNA Polymerase were thawed on ice. All other components were thawed at room temperature. All incubation steps of the protocol were preprogrammed on a thermal cycler.

The resulting cDNA was amplified by real-time qPCR using SYBR Green (Bio-Rad Laboratories) as the fluorophore in a CFX96 real-time thermal cycler (Bio-Rad Laboratories). Specific pairs of primers (Integrated Device Technology) were used for each gene amplification. PCR conditions used were 10 min at 95 °C for 1 cycle, 15 s at 95 °C, 30 s at 60 °C, and 15 s at 72 °C for 40 cycles, followed by a melt curve analysis (0.5 °C every 5 s from 65 to 95 °C). Results were calculated using the 2^-ΔΔCT^ method and expressed as fold changes of expression of genes of interest. All reactions were performed in duplicate, and GAPDH was used as an internal control.

### Data analysis

The animal weights and dietary intake data in Tables 1-3 are presented as means ± SD. Kinetic data are presented as means ± SEM. ANOVA followed by post-hoc comparisons were performed for kinetic data. Two- sided *P* values of 0.05 indicated significance for tests of interactions and main effects. The data were analyzed using GraphPad Prism version 6.0 (GraphPad software, San Diego, CA, USA).

**Table 1:** Effect of low protein diet with and without supplemental folate on whole-body amino acid metabolism in rats. Methionine methyl (Q_m_) and carboxy (Q_c_) fluxes, transsulfuration (TS), transmethylation (TM), homocysteine remethylation (RM), serine flux and cysteine flux in rats

Kinetics (μmol·kg^− 1^·h^− 1^)	Control (*n* = 8)	LP (*n* = 8)	LPF (*n* = 8)	*P* value
Q_c_	97.3 ± 4.9	126 ± 18.9	93.4 ± 13.8	0.22
Q_m_	112 ± 5.8	147 ± 23.7	110 ± 16.1	0.24
Methionine TM	36.0 ± 2.3 ^a^	57.5 ± 4.5 ^b^	56.6 ± 5.8 ^b^	0.003
Methionine TS	21.4 ± 1.5 ^a^	36.6 ± 3.8 ^b^	39.9 ± 4.1 ^b^	0.002
Homocysteine RM	14.6 ± 1.4	20.9 ± 5.0	16.6 ± 2.4	0.40
Serine flux	531 ± 43 ^a^	751 ± 64 ^b^	804 ± 55 ^b^	0.005
Cysteine flux	97.7 ± 7.5	105 ± 5.8	101 ± 6.5	0.75

Note: Data are mean ± SEM; LP = Low Protein; LPF = Low Protein with supplemental folate

Methionine methyl (Q_m_) and carboxy (Q_c_) fluxes, transsulfuration (TS), transmethylation (TM), homocysteine remethylation (RM), serine flux and cysteine flux. Means with different superscript letters are significantly different, *P* < 0.05 (Tukey’s multiple comparison test)

**Table 2:** Effect of low protein diet with and without supplemental folate on erythrocyte and liver glutathione in rats

	Control (*n* = 8)	LP (*n* = 8)	LPF (*n* = 8)	*P* value
Cysteine				
Plasma Concentration (μmol·L^− 1^)	167 ± 5.95 ^a^	180 ± 12.8 ^ab^	218 ± 16.8 ^b^	0.0268
Glutathione				
Erythrocyte				
Concentration (mmol·L^− 1^)	2.97 ± 0.12 ^a^	4.11 ± 0.19 ^b^	4.12 ± 0.35 ^b^	0.003
ASR (mmol·kg^− 1^·h^− 1^)	0.59 ± 0.14	0.91 ± 0.15	0.55 ± 0.13	0.145
Liver				
Concentration (mmol·L^− 1^)	4.00 ± 0.29 ^a^	6.02 ± 0.24 ^b^	5.82 ± 0.40 ^b^	0.0003
ASR (mmol·kg^− 1^·h^− 1^)	4.87 ± 1.13	6.67 ± 1.45	4.45 ± 0.78	0.383

Note: Data are mean ± SEM; LP = Low Protein; LPF = Low Protein with supplemental folate ASR = absolute synthesis rate. Means with different superscript letters are significantly different, *P* < 0.05 (Tukey’s multiple comparison test)

**Table 3:** Effect of low protein diet with and without supplemental folate on oocyte serine and cysteine kinetics and cumulus cell amino acid concentration, respectively, in rats

	Control (*n* = 5)	LP (*n* = 5)	LPF (*n* = 6)	*P* value
Oocyte kinetics (μmol·kg^− 1^·h^− 1^)				
Serine flux	2722 ± 215 ^ab^	2133 ± 191 ^a^	3130 ± 329 ^b^	0.033
Cysteine flux	355 ± 22.9 ^a^	261 ± 25.8 ^b^	255 ± 18.23 ^b^	0.012
TS	416 ± 32.2	274 ± 10.7	387 ± 56.6	0.0796
Cumulus cell amino acid concentration (nmol per nmol pellet phenylalanine)				
Methionine	0.019 ± 0.005 ^a^	0.028 ± 0.008 ^ab^	0.055 ± 0.014 ^b^	0.0434
Homocysteine	0.106 ± 0.015	0.173 ± 0.053	0.217 ± 0.042	0.136
Cysteine	8.94 ± 2.07	9.45 ± 2.25	11.4 ± 3.00	0.765
Serine	4.77 ± 0.89	8.45 ± 2.04	7.72 ± 1.53	0.213
Glycine	7.45 ± 1.42	6.10 ± 0.91	3.68 ± 1.06	0.092

Note: Data are mean ± SEM; LP = Low Protein; LPF = Low Protein with supplemental folate

*TS* transsulfuration (conversion of serine to cysteine)

Means with different superscript letters are significantly different, *P* < 0.05 (Tukey’s multiple comparison test)

## Results

### Amino acid kinetics

**Fig. 2:**
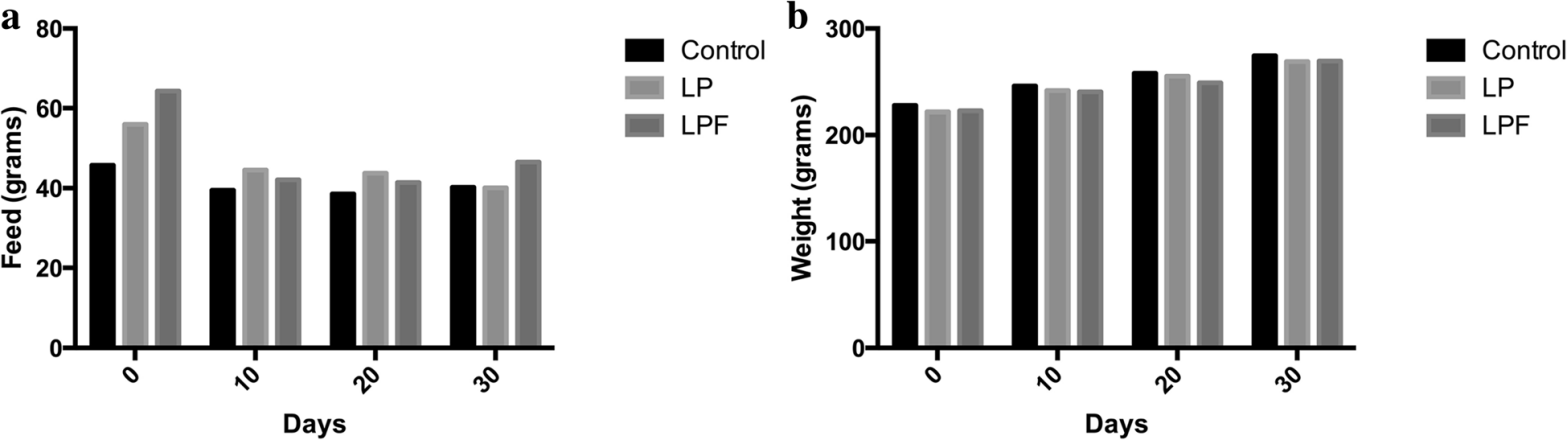
Average feed consumed and pup weights (**a**, **b**)

The animals were allowed unlimited access to food and water across all experimental dietary groups. The daily average feed consumed and the daily average pup weights were not statistically different among the three experimental groups (Figs. 2a-b). Each of the three isocaloric dietary formulations (control, LP, LPF) contained 3.8 Kcal/g of feed. Since the amount of feed intake was not significantly different among the three groups, the energy consumed was also not significantly different between the three dietary groups throughout the 40 day dietary intervention. In the LP dietary group, 6.5% of Kcal consumed were derived from protein. Next, components of TM and TS were investigated, including metabolic flux, which describes the rate of turnover of molecules within the metabolic pathway. Plasma methionine TM to S-adenosyl homocysteine (SAMe) was increased in the LP and LPF groups. Plasma methionine TS was also increased in the LP and LPF groups, indicating that the methionine cycle was turning over more rapidly as both the transfer of methyl groups to acceptors and sulfur groups to cysteine were occurring at a faster rate (Table 1). The flux of serine was also increased in the LP and LPF groups, indicating that the transfer of methyl groups from homocysteine to cystathionine, a critical step in the production of glutathione, was increased (Table 1).

There was a significantly higher plasma cysteine concentration in the LPF group compared to the control group which might ultimately support a more rapid synthesis of glutathione via TS with supplemental folate. This is further supported by a higher concentration of glutathione both in red blood cells and in the liver of LP and LPF animals (Table 2). The absolute synthesis rate of glutathione in both red blood cells and in the liver also trended higher toward significance in the LP group when compared to the control and LPF groups (Table 2).

Amino acid kinetics was then examined specifically within the cumulus oocyte complex. Within the oocyte proper, the flux of serine was lower in the LP group compared to the LPF group (Table 3). The flux of cysteine was decreased in the LP and LPF groups compared to the control group (Table 3). The de novo synthesis of cysteine from the TS of methionine combining with serine demonstrated a decrease that trended toward significance in the LP group compared to the control and LPF groups (Table 3). There were no significant differences detected in the concentrations of amino acids among the groups in pooled groups of cumulus cells.

The cumulus cells were denuded from around the oocyte, purified, and analyzed separately. The concentration of methionine in the LPF group was significantly higher than in the control group, suggesting an increased availability of this amino acid in the folic acid supplemented group (Table 3). The concentrations of homocysteine and serine trended higher in both the LP and LPF groups when compared to the control group. Conversely, the concentration of glycine trended lower in the LP and LPF groups when compared to the control group. Together, these patterns in the cumulus cells suggest that protein restriction causes altered amino acid metabolism, with improved methionine availability in the LPF group when compared to both the control and low protein groups.

In summary, with regard to amino acid kinetics within the rat, preovulatory dietary protein restriction increased transmethylation, transsulfuration and the flux of serine within the plasma, reflecting altered whole body metabolism. Within the oocyte proper, serine flux was decreased in the LP compared to the LPF group, and cysteine flux was decreased in the LP and LPF groups compared to the control group, suggesting a reduced availability of serine to provide methyl groups to tetrahydrofolate (THF) to form 5-methyl-THF, an intermediate in the conversion of homocysteine to methionine. Additionally, protein restriction increased the concentration of glutathione in both red blood cells and in the liver in LP and LPF groups compared to the control group (*p* = 0.003 and *p* = 0.0003). Serine is necessary for the provision of methyl groups for the formation of metabolic intermediates as well as the methylation of DNA, RNA and protein, and a reduced flux in the oocyte when exposed to protein restriction may affect reproductive competency of the cell.

### Cumulus oocyte complex mitochondrial ultrastructure

**Fig. 3:**
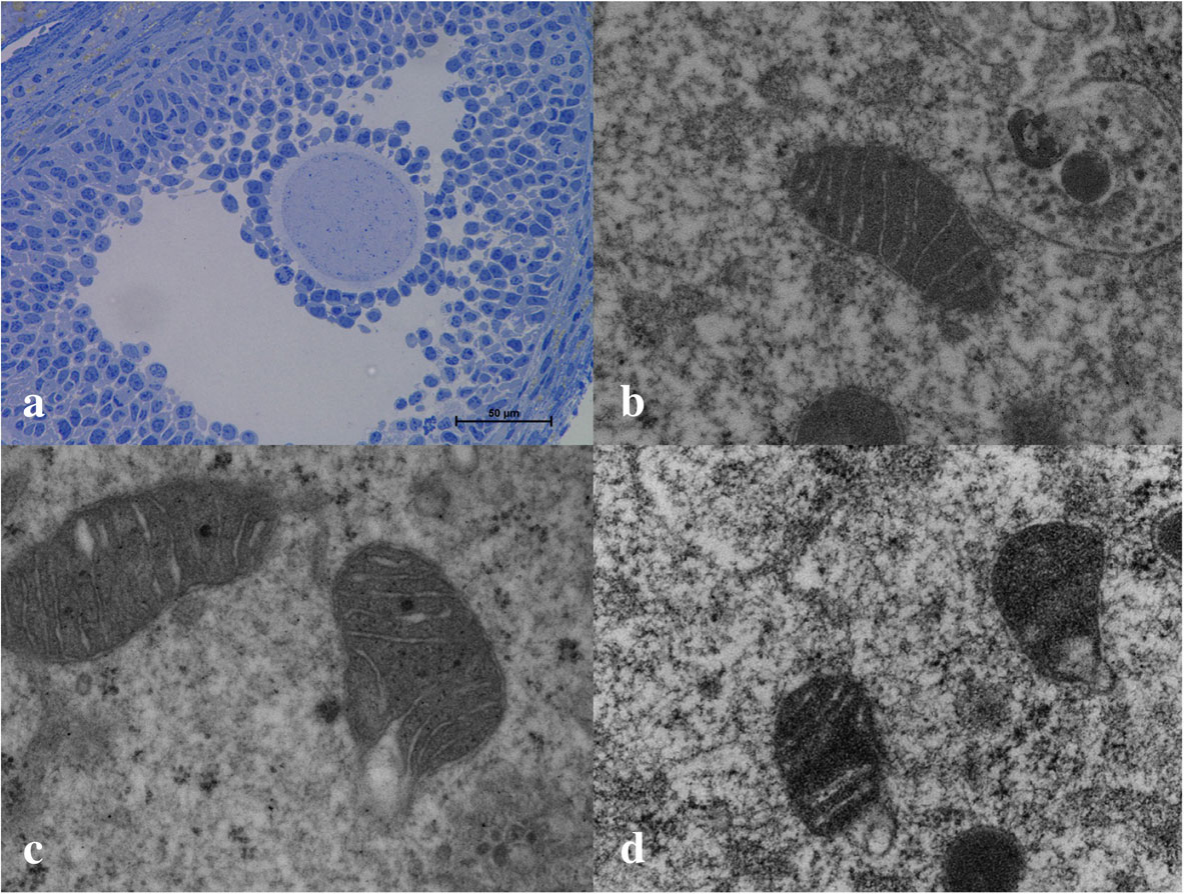
Cumulus oocyte complex at 40x (**a**), control oocyte mitochondrion at 2500x (**b**), LP oocyte mitochondrion at 2500x (**c**), and LPF mitochondrion at 2500x (**d**)

Here the mitochondria of oocytes and cumulus cells in antral follicles were characterized in a semi-quantitative manner by assessing the prevalence of ultrastructural features that have previously been correlated with abnormal mitochondrial function. Antral follicles were identified under light microscopy (Fig. 3a). Mitochondrial ultrastructure in oocytes and cumulus cells was observed utilizing transmission electron microscopy.

Oocytes in the LP and LPF groups demonstrated an increased prevalence of abnormal mitochondria (*p* < 0.01 and *p* < 0.05, respectively) compared to oocytes in the control group (control = 14.6%, LP = 52.7%, LPF = 47.1%, Figs. 3b-d). Cumulus cells in the LP and LPF groups also demonstrated an increased prevalence of abnormal mitochondria compared to the control group (*p* < 0.001 and *p* < 0.05, respectively). The proportion of abnormal mitochondria in the cumulus cells was 12.7% in the control group, 52.4% in the LP group and 38.9% in the LPF group.

### Oocyte mRNA expression

**Fig. 4:**
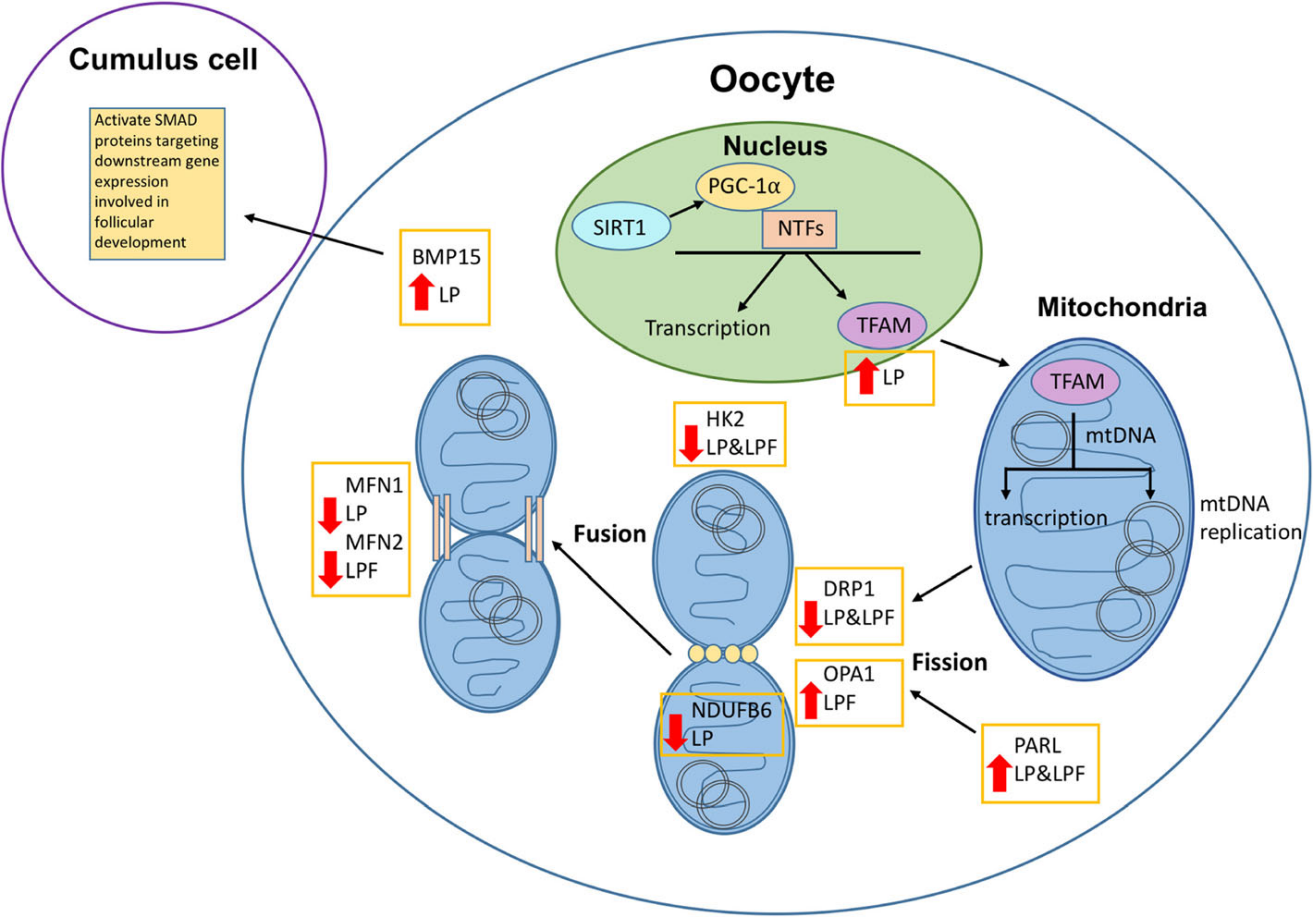
Effects of protein restriction on the transcriptional regulation of mitochondrial biogenesis

The transcriptional control of mitochondrial biogenesis is complex and involves the activation of nuclear transcription factors. Regulation of mitochondrial fission and fusion results in appropriate mitochondrial network organization. The *mRNA* expression of 18 genes involved in mitochondrial biogenesis and metabolism were quantified in pooled oocytes from control, LP and LPF groups of rats, and 9 of these genes were differentially regulated in the protein restricted groups, including *Bmp15, Drp1, Hk2, Mfn1, Mfn2, Ndufb6, Opa1, Parl* and *Tfam* (Fig. 4). *Gapdh* was used as the housekeeping gene for all PCR experiments.

**Fig. 5:**
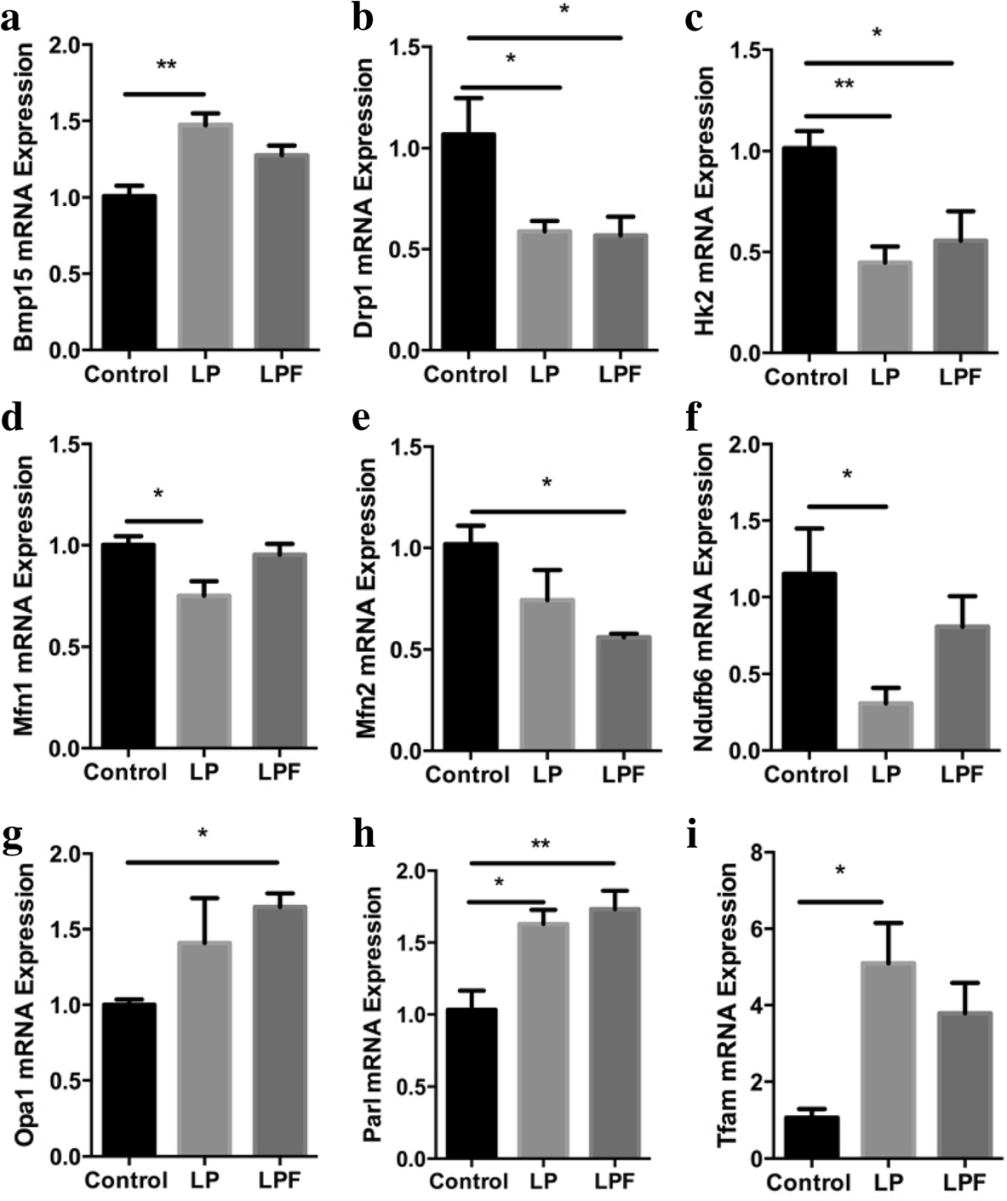
Oocyte *m*RNA expression of genes related to mitochondrial biogenesis and function. * *p* < 0.05, ** *p* < 0.01

*Bmp15, Parl,* and *Tfam m*RNA expression were increased in the LP group compared to the control group. *Drp1, Hk2, Mfn1* and *Ndufb6 m*RNA expression were decreased in the LP group compared to the control group. *Opa1 m*RNA expression was increased in the LPF group, and there was a trend toward increased expression in the LP group, compared to the control group. *Mfn2* expression was decreased in the LPF group, and there was a trend toward decreased expression in the LP group, compared to the control group (Figs. 5a-i).

## Discussion

It is remarkable that the oocyte, as a precursor to human life, can thrive in an arguably fragile environment representing a watershed of nutrient access during maturation and early embryonic growth. Maternal dietary intake critically supports the oocyte through maturation, fertilization and until embryo genome activation. With high-energy demands, a limited pool of mitochondria and a reduced capability of protecting itself from oxidative damage, the oocyte must adapt to its host environment to survive – potentially at significant future costs to offspring. The oocyte, embryo and fetus alike may be exposed to metabolic stressors during development. Extensive data studying the effects of the Dutch famine that occurred during the winter of 1944–45 indicate that gestational exposure to maternal undernutrition can have lasting effects on offspring health. Specifically, glucose intolerance, obesity, coronary heart disease, and altered lipid profiles were seen in 2414 people born during or after the Dutch famine [53]. Adverse health effects were more severe in offspring the earlier the exposure during development. Other observations from offspring conceived during the Dutch famine suggest increased risk of schizophrenia and depression with worse cognitive task performance, possibly due to altered aging mechanisms [54]. Interestingly, observations from the Chinese famine of 1959–62 indicate a sex-specific association with metabolic syndrome, with fetal and childhood-exposed women having a higher prevalence of metabolic syndrome compared to non-exposed [55].

The maternal-zygotic transition is a sensitive stage in human development that is poorly understood. Maternal dietary intake is the only source of sustenance for the oocyte and early embryo [7]. Nutrient availability prior to ovulation must support fertilization, unpacking of the paternal genome and early cleavage of the zygote until embryo genome activation begins. Protein synthesis in the human increases 38-fold during the growth phase of oocytes [56], and growing follicles are located in the well-vascularized corticomedullary border, facilitating nutrient exchange in the growing oocyte [57]. Gap junctions between the oocyte and granulosa cells allow free transit of small molecules [58]. Amino acids within the ovary and early embryo serve as substrates necessary for DNA and organelle synthesis. Production of the antioxidant glutathione helps prevent cellular damage, especially in mitochondria, and accumulates during oocyte growth and maturation.

Integral to the optimization of intracellular processes is the regulation of the transfer of methyl groups for the synthesis of metabolic intermediates and the methylation of DNA, RNA and proteins. Serine and glycine are nutritionally non-essential amino acids that are critical for proper cell function. Serine provides the carbon skeleton for the synthesis of cysteine from methionine, and is additionally a precursor for glycine synthesis, one of the amino acids that forms glutathione (Fig. 1). Serine provides methyl groups to tetrahydrofolate (THF) to form 5-methyl-THF, which is an intermediate in the conversion of homocysteine to methionine. Therefore, the availability of methionine is in part a reflection of resynthesis from homocysteine. The methylation of homocysteine to methionine requires an adequate supply of folic acid. Through the TS pathway, serine combines with homocysteine to produce cystathionine, and ultimately cysteine, which is the rate-limiting substrate for the production of the major intracellular antioxidant glutathione. Single carbon transfers are critical for normal cellular function and require the availability of necessary precursors such as the amino acids serine, glycine, and methionine. Serine and glycine are nutritionally non-essential for humans, but methionine is nutritionally essential. Folic acid derivatives are critical for the remethylation of homocysteine to methionine, and in the setting of protein restriction (limiting access to methionine), folic acid may provide the cell a mechanism for compensation.

Preovulatory protein restriction in this model resulted in significant changes in amino acid metabolism (within the plasma and also within specific tissues such as the liver and oocyte), in cumulus oocyte complex mitochondrial ultrastructure and in the *m*RNA expression of mitochondrial biogenesis and metabolism related genes in the rat. Preovulatory dietary protein restriction, in plasma, increased methionine transmethylation, transsulfuration, and serine flux. This suggests that the rate of amino acid turnover is increased when the absolute availability of these precursors is decreased. Protein restriction also increased the concentration of glutathione in erythrocytes and in liver. Together, it is hypothesized that the decreased availability of GSH precursors during protein restriction leads to metabolic compensation; that is, increased methionine TM, TS, and serine flux. This allows for an increased concentration of GSH during periods of perceived increased ROS exposure. This may protect the organism from ROS-induced damage during dietary protein restriction. The effects of folic acid supplementation had differential effects in the plasma when compared to the oocyte. In plasma, supplemental folic acid in the setting of protein restriction did not appear to ameliorate the observed metabolic effects.

Preovulatory protein restriction in the oocyte demonstrated differential metabolic effects. The flux of serine was suppressed in the LP group compared to the LPF group, and transsulfuration trended lower in the LP group compared to the LPF and C groups, suggesting that the oocyte is unable to increase antioxidant precursor production in a state of protein starvation. Cysteine flux was also suppressed in the LP and LPF groups compared to the control group. Importantly, oocyte serine flux and transsulfuration demonstrated reversal of LP changes when supplemented with folic acid. This suggests that folic acid supplementation allowed for partial normalization of the observed effects within the oocyte. Future studies will focus on embryonic effects of low protein exposure during the preovulatory period. This will be done via mating of protein-restricted female rats prior to and during ovulation with control male rats to assess embryonic and fetal development.

It may be inferred that oocytes exposed to reduced protein availability are vulnerable to intracellular oxidative stress as they cannot increase glutathione production. ROS are formed locally by mitochondria as a byproduct of the formation of ATP, capable of inducing oxidative damage to mitochondrial DNA (mtDNA) [59]. Compared to nuclear DNA, mtDNA is more susceptible to mutation due both to its proximity to ROS as well as a reduced capacity for DNA repair [32]. Increased mtDNA mutations may limit normal mitochondrial function, leading to decreased energy production and a subsequent inability to support increasing intracellular demands. Structural mitochondrial abnormalities have been documented after exposure to starvation and other models of cellular stress and nutritional deficiency [15, 35]. Observed metabolic aberrations in the oocyte exposed to protein restriction, including reduced serine flux and trend toward reduced transsulfuration, may indicate an inability within the oocyte to increase glutathione precursors and may contribute to the ultrastructural changes that were seen in cumulus oocyte mitochondria. Abnormal mitochondria that are inherited by offspring may precipitate adverse health effects in the future, potentially related to metabolic syndrome, aging, fertility, and cardiovascular disease. Folic acid did not rescue the observed adverse effects in mitochondrial ultrastructure. Cumulus oocyte complexes have high cellular energy demands and structurally abnormal mitochondria may affect the reproductive potential of oocytes. Further, dietary protein restriction during follicular development altered *m*RNA expression patterns of many genes related to mitochondrial biogenesis and metabolism within the rat oocyte. Supplemental folate did not appear to reliably ameliorate these changes in gene transcription.

Genes that were differentially expressed in the protein-restricted groups included *Bmp15, Drp1, Hk2, Mfn1, Mfn2, Ndufb6, Opa1, Parl* and *Tfam*. Expression of *Drp1* in pooled oocytes was significantly lower in the LP and LPF groups compared to the control group and this potentially reflects altered fission of the outer mitochondrial membrane. Trends in *Mfn1* and *Mfn2* expression, that were decreased in the LP and LPF groups, respectively, suggest potentially inhibited downstream mitochondrial fusion mechanisms. *TFAM* activates transcription and replication of the mitochondrial genome and oocyte *m*RNA expression of *Tfam* was significantly increased in the LP group compared to the control group, suggesting increased mtDNA replication in a protein restricted environment. *Bmp15* is involved in oocyte maturation and follicular development via paracrine signaling with cumulus cells that is mediated by SMAD activation and downstream gene target activation [60]. SMAD proteins are responsible for the signal transduction of receptors of the transforming growth factor beta superfamily. Defects in Bmp15 are implicated in cases of ovarian dysgenesis. The *m*RNA expression of *Bmp15* was significantly increased in the LP group compared to the control group, suggesting that ovarian function may be altered with protein restriction. These altered *m*RNA expression patterns suggest that protein restriction may alter not only mitochondrial ultrastructure in cumulus oocyte complexes but also, ultimately, function.

Future studies will focus on the impact of preovulatory protein restriction and folate supplementation on embryonic development. Specifically, we will study in vitro early embryonic morphokinetic parameters utilizing EmbryoScope time lapse microscopy in embryos up to the hatching blastocyst stage. Subsequent studies will focus on the impact of dietary protein restriction on late gestational and postnatal offspring development.

## Conclusions

This series of experiments provides metabolic, ultrastructural and transcriptional evidence that protein restriction during follicular development adversely affects the growth and development of cumulus oocyte complexes in a rat model. In the whole body, protein restriction preferentially shunted nutrients toward the production of antioxidant precursors. Within the oocyte proper, protein restriction preferentially shunted nutrients toward the remethylation of methionine, away from the production of antioxidant precursors. Folic acid may be able to rescue some of the observed metabolic effects within the oocyte. With high energy demands, a limited pool of mitochondria and a reduced capability of protecting itself from oxidative damage, the oocyte must adapt to its host environment – potentially at significant future costs to offspring.
